# Disparities in the Outcomes Following Ischemic Stroke Between the Floating Population and Indigenous Population of Shanghai

**DOI:** 10.3389/fneur.2021.774337

**Published:** 2021-12-15

**Authors:** Xiaochuan Liu, Qian Sun, Sichen Yao, Junhui Zhang, Huanyin Li

**Affiliations:** ^1^Department of Neurology, Minhang Hospital, Fudan University, Shanghai, China; ^2^Wujing Community Health Service Center, Shanghai, China; ^3^Nanqiao Community Health Service Center, Shanghai, China

**Keywords:** floating population, ischemic stroke, prognosis, health care, Shanghai

## Abstract

**Background and Purposes:** Through this study, we hope to gain more insights into the differences in outcome following an ischemic stroke between the floating population and the indigenous population of Shanghai.

**Method:** In this retrospective cohort study, we analyzed patients with first-ever acute ischemic stroke who were admitted to a comprehensive stroke center in the Minhang district, Shanghai, from January 1, 2019, to December 31, 2020. All patient's demographic data and medical histories were prospectively collected and they were followed up for at least 3 months. The Indigenous population of Shanghai was defined as patients with an identification number starting with 310. All others were treated as floating population. The primary outcome was defined as an unfavorable prognosis at 3 months, with a modified Rankin Scale (mRS) score above 1. Secondary outcomes included the use of emergency medical service (EMS), 3 h arrival rate, and endovascular therapy in eligible patients. Logistic regression analysis was applied to investigate the differences.

**Results:** Finally, 698 patients with first-ever acute ischemic stroke were included (with mean age of 65.32 years, 74.6% men). Of these, 302 patients belonged to the floating population group. Indigenous populations with ischemic stroke were older than the floating population (68.26 years vs. 61.47 years, *P* < 0.001). The floating population was more likely to achieve favorable outcomes at 3 months compared with the indigenous population in multivariable logistic regression analysis [Odds ratio (OR): 0.49, 95% CI: 0.32–0.75, *P* = 0.001]. The use of EMS, 3 h arrival rate, and the application of endovascular therapy were comparable between the floating population and indigenous population (OR: 0.89, 95% CI: 0.62–1.27, *P* = 0.519; OR: 0.78, 95% CI: 0.56–1.09, *P* = 0.14; and OR: 0.82, 95% CI: 0.54–1.26, *P* = 0.365, respectively).

**Conclusion:** Compared with the indigenous population, the floating population with the first-ever ischemic stroke was more likely to have a favorable outcome at 3 months.

## Introduction

With the fast growing economy of China, an increasing number of workers from rural areas choose to seek job opportunities in first-tier cities like Beijing, Shanghai, and Shenzhen (1). These workers are usually called the floating population, which means that although they work in big cities, they would return to the place where they were born to meet their family members during traditional holidays such as the Spring Festival holiday. The unique household registration (Hukou) system contributed partly to the formation of the floating population. Some studies also refer to them as migrant workers (2–4). The seventh Chinese census indicated that there are ~24 million individuals living in Shanghai, and the floating population accounts for nearly half of them (5). The health of the floating population was reported to be associated with many aspects such as social insurance status, occupational exposure, and acculturative stress (6–8). Previous studies have found that there exists a higher chance of the floating population being diagnosed with diseases that are more likely work-related (3, 9). However, the impact of the floating population on cardiovascular diseases such as ischemic stroke has rarely been investigated. As one of the leading causes of death and acquired disability in our country (10, 11), it is worth exploring whether behaviors associated with seeking medical help (e.g., prehospital delay and calling the ambulance) after ischemic stroke were different between the floating population and indigenous population. Furthermore, it is unknown whether there are differences in the short-term outcome following a first-ever ischemic stroke between these two groups. Through this study, we hope to gain more insight into these aspects.

## Methods

### Study Design and Population

In this retrospective cohort study, we analyzed patients with first-ever ischemic stroke, who were admitted to our stroke center between January 2019 and December 2020. Our hospital is the central hospital of the Minhang district located in suburban Shanghai and is also a comprehensive stroke center. The clinical information of our patients was prospectively collected from our database, and the study was approved by the institutional review board affiliated with Fudan University (2020-057-01k). Written informed consent was obtained from all the patients or their welfare guardians for data collection and subsequent analysis. The data that support the findings of this study may be available from the corresponding author under reasonable request.

### Data Collection

We retrieved all patient data collected from January 1, 2019, to December 31, 2020, from our online database. Only patients with first-ever ischemic stroke admitted within 24 h were included in this analysis. Researches of our team verified the data by checking their original clinical data recorded in our system. Ischemic stroke in all patients was confirmed by computed tomography (CT) or magnetic resonance imaging (MRI). The prehospital delay was defined as the time between symptom onset and arrival of the patient at the hospital. As for patients with unknown symptom onset time, we defined the time of last known to be well as the onset time. Patients with hemorrhage stroke, stroke mimic, or previous ischemic stroke were excluded from this study. Demographic data and previous medical histories were included in the analysis. The ischemic stroke subtype was routinely classified according to the Trial of Org 10172 in Acute Stroke Treatment (TOAST) (12) criteria by neurologists at our center. Stroke severity was assessed on admission using the NIH Stroke Scale (NIHSS). Risk factors such as alcohol consumption and smoking habit were defined as binary variables (0 = No; 1 = Yes). We assigned 0 (No) to the patients who had never smoked or consumed alcohol and those who had quit for more than 1 year. Medical histories such as hypertension and atrial fibrillation (AF) were defined according to standard clinical criteria and were confirmed within 24 h of admission. Endovascular therapy included intravenous thrombolytic therapy (IVT) and intravascular thrombectomy. The outcome at 3 months was evaluated *via* modified Rankin Scale (mRS), and it was assessed through out-patient routine visits or structured telephonic interviews by experienced neurologists or trained nurses who were blind to patient archives. Patients lost to follow-up were excluded from the analysis.

### Definition of Floating Population

Although there are several different definitions for the floating population in previously published studies (13, 14), the most commonly used one is “individuals who have lived at a destination for at least 6 months (15).” Since this is a retrospective study, we could not find any information of our patients with regard to their duration of stay in the Minhang district, Shanghai; however, the database contained patient identification numbers and place of residence. Each person has a unique identification number assigned to them at birth. This number can be used to identify whether the person is local to the district or not. For example, the identification number of local born Shanghai residents starts with 310. Accordingly, if a patient has an identification number that starts with numbers other than 310, we can conclude he is not a local born resident; in other words, he is not an indigenous population. In this study, we defined a floating population as patients with an identification number starting with numbers other than 310.

### Definition of Primary and Secondary Outcomes

The primary outcome was defined as an unfavorable prognosis at 3 months, with an mRS score above 1. Secondary outcomes included the use of emergency medical service (EMS), 3 h arrival rate, and the application of endovascular therapy among eligible patients.

### Sensitivity Analysis

To confirm whether our findings were applicable to patients with ischemic stroke who seek medical services beyond 24 h, we replicated our analyses among those patients admitted to our stroke center within 3 days.

### Statistical Analysis

Baseline characteristics were compared by Fisher's exact test for categorical variables, and a two-sample *t*-test for continuous variables. Between-group differences in primary and secondary outcomes were investigated by logistic regression analysis. Differences in mRS categorical data at 3 months were compared with Fisher's exact test. Confounders including age, gender, smoking history, drinking habit, hypertension, diabetes, dyslipidemia, atrial fibrillation, NIHSS at admission, recanalization therapy, TOAST, and discharge mRS were adjusted in multivariable logistic regression analysis. Odds ratio (OR) and 95% CI were calculated. To test the interaction effect of the floating population on our primary outcome across several pre-defined subgroups (defined based on an age cutoff of 60 years, gender, smoking history, drinking habit, hypertension, diabetes, dyslipidemia, atrial fibrillation, and minor stroke defined as NIHSS at admission ≤ 3), we tested the statistical significance of the variable floating population/indigenous population × subgroup in a multivariable logistical regression model. The statistical analysis was performed on STATA (Version 15.0 Stata Corp College Station, Texas, USA). R software (R version 3.5.3 The R Foundation for Statistical Computing) was used in creating the forest-plot. A two-tailed *P*-value < 0.05 was considered statistically significant.

## Results

### Study Flow

**Figure 1:**
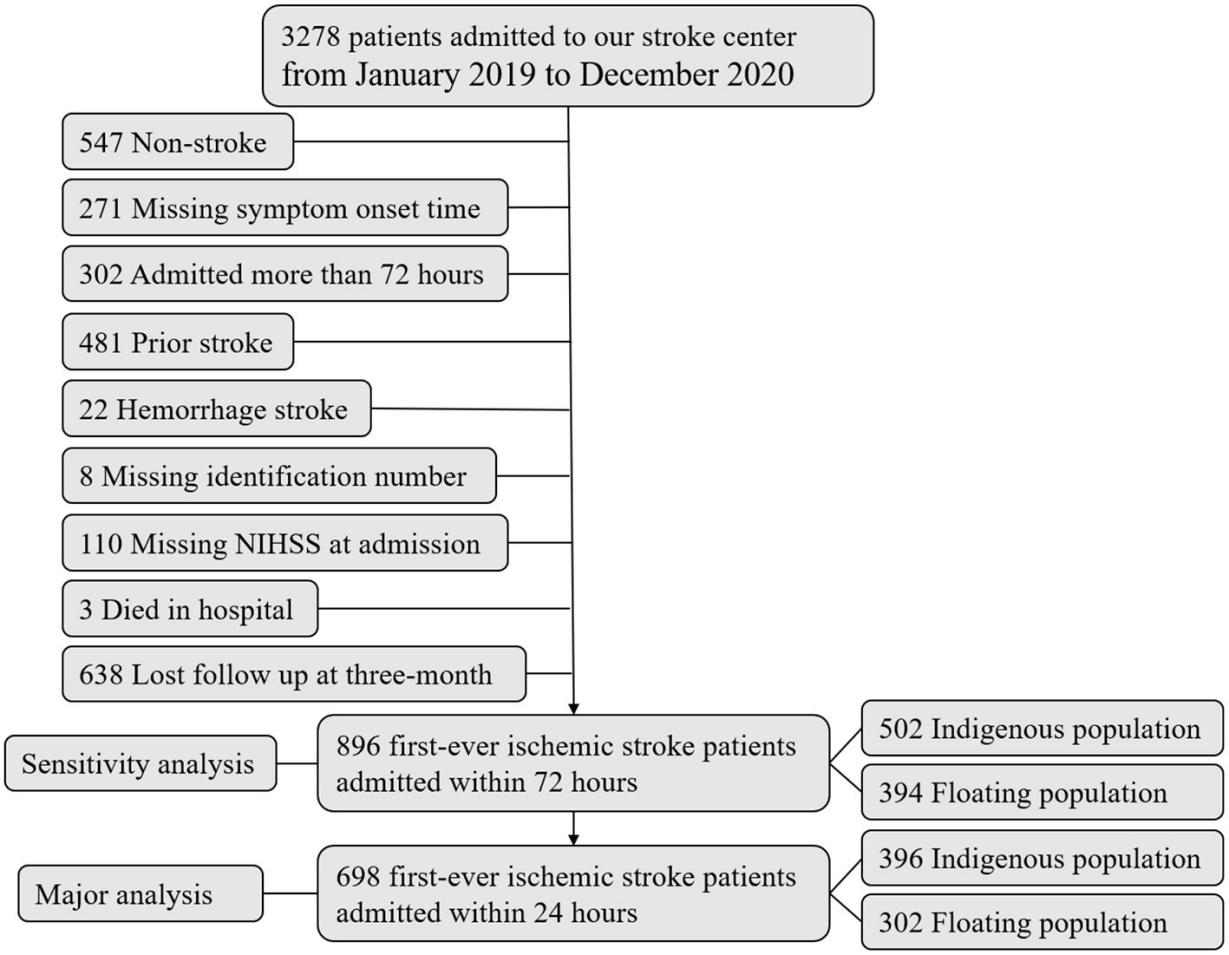
Study flowchart. NIHSS, NIH Stroke Scale.

Figure 1 shows us the study flowchart. There were 3,278 patients admitted to our stroke center in the past 2 years from January 1, 2019, to December 31, 2020. Briefly speaking, we excluded 2,580 patients who were not satisfied with the aim of this study. Finally, 698 patients with first-ever ischemic stroke, with onset-to-admission within 1 day, were included in our final analysis. Significant differences were found in most baseline characteristics between the included and excluded patients (Supplementary Table I).

### Baseline Characteristics of the Study Population

Table 1 shows the baseline characteristics of patients with ischemic stroke admitted within one day in this study. The mean age was 65.32 years (74.6% men). Further, 396 patients belonged to the indigenous population with an identification number starting with 310, and the others comprised the floating population. The top three places where the floating population came from were Anhui Province, Jiangsu Province, and Henan Province (Supplementary Figure I). Indigenous population with ischemic stroke were older than the floating population with ischemic stroke (68.26 years vs. 61.47 years, *P* < 0.001). The proportion of men was higher in the floating population than in the indigenous population (79.8 vs. 70.7%, *P* = 0.007). The prehospital delay was shorter in the indigenous population with ischemic stroke than in the floating population (median 3.78 h vs. 5.32 h, *P* = 0.043). However, the indigenous population with ischemic stroke was more likely to have been diagnosed with diabetes compared with the floating population (39.4 vs. 27.2%, *P* < 0.001).

**Table 1:** Baseline characteristics of patients with acute ischemic stroke.

**Factor**	**All**	**Floating population**	**Indigenous population**	***p*** **-value**
***N***	**698**	**302**	**396**	
Age, mean (SD)	65.32 (11.19)	61.47 (11.36)	68.26 (10.13)	<0.001
Gender (Male)	521 (74.6%)	241 (79.8%)	280 (70.7%)	0.007
Prehospital delay (median, IQR, hour)	4.58 (1.5, 11)	5.32 (1.62, 11.78)	3.78 (1.38, 10.05)	0.043
Alcohol consumption	97 (13.9%)	48 (15.9%)	49 (12.4%)	0.19
Smoking history	213 (30.5%)	102 (33.8%)	111 (28.0%)	0.11
**Medical histories**				
Hypertension	463 (66.3%)	197 (65.2%)	266 (67.2%)	0.63
Diabetes	238 (34.1%)	82 (27.2%)	156 (39.4%)	<0.001
Dyslipidemia	196 (28.1%)	93 (30.8%)	103 (26.0%)	0.17
Atrial fibrillation	81 (11.6%)	29 (9.6%)	52 (13.1%)	0.15
NIHSS at admission (median, IQR)	3 (1, 5)	3 (1, 5)	3 (1, 5)	0.72
Discharge mRS	2 (1, 3)	2 (1, 3)	2 (1, 3)	0.36
Intravenous thrombolytic therapy	129 (18.5%)	55 (18.2%)	74 (18.7%)	0.92
Intravascular thrombectomy	28 (4.0%)	11 (3.6%)	17 (4.3%)	0.70
TOAST				0.43
Large-artery atherosclerosis	276 (39.5%)	117 (38.7%)	159 (40.2%)	
Cardioembolism	60 (8.6%)	22 (7.3%)	38 (9.6%)	
Small vessel disease	289 (41.4%)	134 (44.4%)	155 (39.1%)	
Other etiology	20 (2.9%)	10 (3.3%)	10 (2.5%)	
Unknown etiology	53 (7.6%)	19 (6.3%)	34 (8.6%)	

*NIHSS, NIH Stroke Scale; IQR, interquartile range; P-values represent the differences between the indigenous population and floating population; mRS, modified Rankin Scale*.

### The Impact of Floating on Patients With Ischemic Stroke

**Figure 2:**
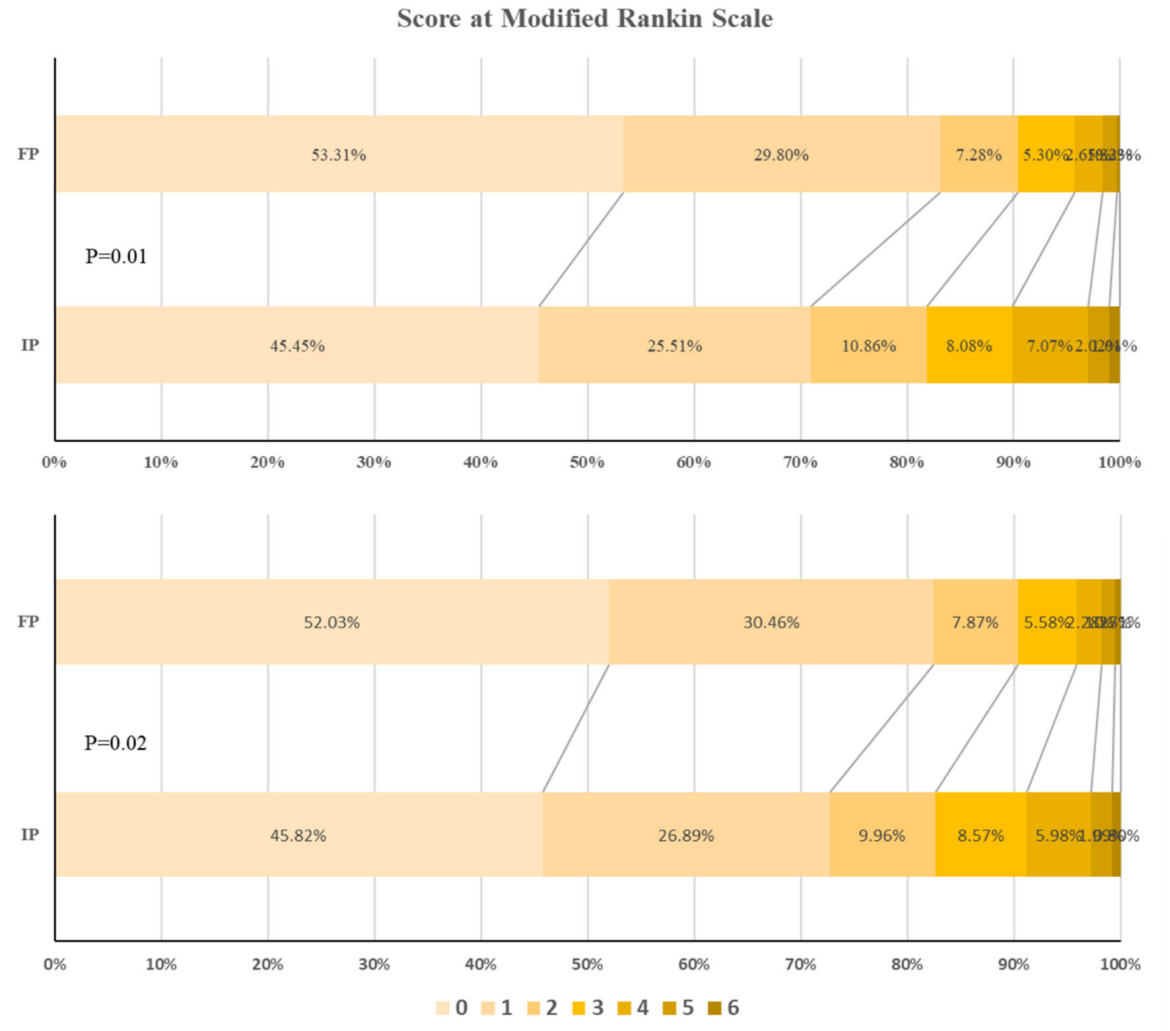
The outcome at 3 months based on modified Rankin Scale (mRS) score. IP, indigenous population; FP, floating population. Top, Among patients with acute ischemic stroke. Bottom, Among patients with subacute ischemic stroke (sensitivity analysis).

Table 2 shows us the impact of floating on the primary and secondary outcomes. The floating population with ischemic stroke was more likely to achieve favorable outcomes at 3 months compared with the indigenous population in both the univariable logistic regression analysis (OR: 0.50; 95% CI: 0.34–0.72, *P* < 0.001) and multivariable logistic regression analysis (OR: 0.49; 95% CI: 0.32–0.75, *P* = 0.001). There were nearly 50% higher chances of achieving better outcomes in the floating population with ischemic stroke than in the indigenous population. We also compared the outcome at 3 months based on mRS categorical data in a bar graph format (Figure 2). The proportion of ischemic stroke patients with favorable outcomes at 3 months is significantly higher among the floating population. With regard to the proportion of patients with ischemic stroke who arrived at the hospital *via* ambulance, non-statistical differences were observed between the floating population and indigenous population (32.8 vs. 37.4%, OR: 0.89; 95% CI: 0.62–1.27, *P* = 0.519). Although the indigenous population with ischemic stroke tended to have a shorter prehospital delay, the between-group differences in 3 h arrival rate were non-significant (37.4% vs. 43.7%, OR: 0.78; 95% CI: 0.56–1.09, *P* = 0.146). The rate of achieving endovascular therapy was also not significant between floating population and indigenous population (19.2 vs. 21.5%, OR: 0.82; 95% CI: 0.54–1.26, *P* = 0.365).

**Table 2:** The impact of floating on patients with acute ischemic stroke.

	**Indigenous population**	**Floating population**	***p*** **-value**	**Unadjusted**	***p*** **-value**	**Adjusted**	***p*** **-value**
	***N*** **= 396 (Reference)**	***N*** **= 302**		**OR**		**OR**	
**Primary outcome**							
Unfavorable outcome	115 (29.0%)	51 (16.9%)	**<0.001**	0.50 (0.34–0.72)	**<0.001**	0.47 (0.30–0.74)	**0.001** ^**a**^
**Secondary outcome**							
Emergency medical service	148 (37.4%)	99 (32.8%)	0.23	0.82 (0.60–1.12)	0.209	0.89 (0.62–1.27)	0.519^b^
3-h arrival	173 (43.7%)	113 (37.4%)	0.10	0.77 (0.57–1.05)	0.095	0.78 (0.56–1.09)	0.146^b^
Endovascular therapy	85 (21.5%)	58 (19.2%)	0.51	0.87 (0.60–1.26)	0.464	0.82 (0.54–1.26)	0.365^b^

*OR, odds ratio; CI, confidence interval; Endovascular therapy included intravenous thrombolytic therapy and intravenous thrombectomy; In the multivariable logistic regression analysis, as for a: we adjusted age, gender, emergency medical service, prehospital delay, smoking history, drinking habit, NIHSS at admission, hypertension, diabetes, atrial fibrillation, dyslipidemia, intravenous thrombolytic therapy, intravenous thrombectomy, TOAST, and discharge mRS; as for b: we adjusted age, gender, smoking history, drinking habit, NIHSS at admission, hypertension, diabetes, atrial fibrillation, and dyslipidemia. Bold values mean statistically significant*.

**Figure 3:**
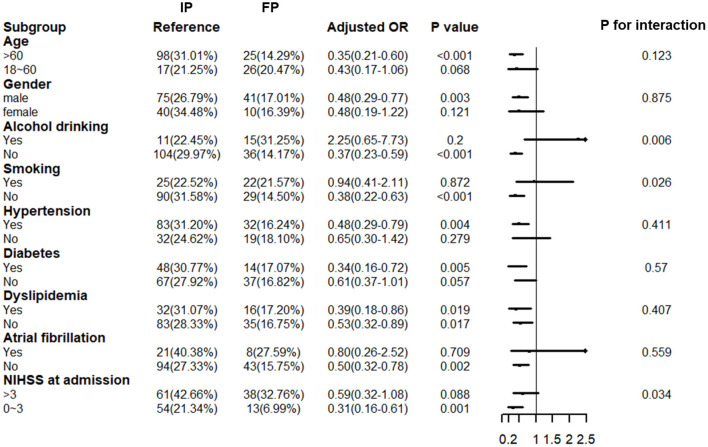
Interactive effects of floating across subgroups for the primary outcome. IP, indigenous population; FP, floating population; OR, odds ratio; CI, confidence interval; NIHSS, NIH Stroke Scale; In the multivariable logistic regression analysis, we adjusted age, gender, emergency medical service (EMS), prehospital delay, smoking history, drinking habit, NIHSS at admission, hypertension, diabetes, atrial fibrillation, dyslipidemia, intravenous thrombolytic therapy, intravenous thrombectomy, and TOAST.

As we observed a favorable outcome for the floating population with ischemic stroke at 3 months, we tested whether there existed interactive effects within different subgroups. Figure 3 shows the interactive effects of floating in several subgroups. Significant interactive effects were found in subgroups of smoking, drinking, and NIHSS at admission (*P* = 0.026, 0.006, and 0.034, respectively). The effect was more apparent in the floating population with no drinking habit (OR: 0.37; 95% CI: 0.23–0.59, *P* < 0.001), no smoking history (OR: 0.38; 95% CI: 0.22–0.66, *P* < 0.001), and minor stroke (OR: 0.31; 95% CI: 0.16–0.61, *P* < 0.001).

### Sensitivity Analysis

**Figure 4:**
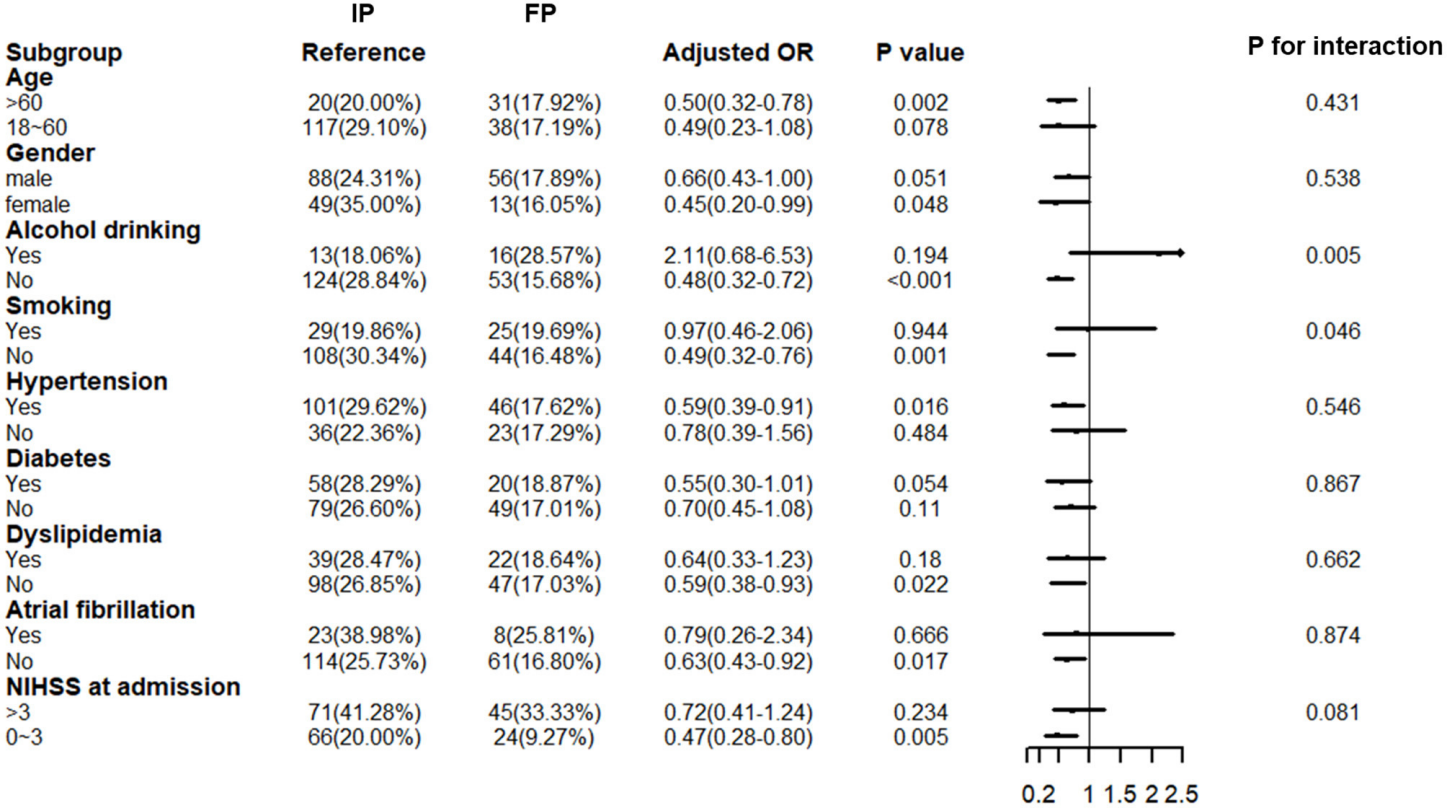
Interactive effects of floating across subgroups for primary outcome among patients with subacute ischemic stroke (sensitivity analysis). IP, indigenous population; FP, floating population; OR: odds ratio; CI, confidence interval; NIHSS, NIH Stroke Scale; In the multivariable logistic regression analysis, we adjusted age, gender, EMS, prehospital delay, smoking history, drinking habit, NIHSS at admission, hypertension, diabetes, atrial fibrillation, dyslipidemia, intravenous thrombolytic therapy, intravenous thrombectomy, and TOAST.

A total of 896 patients with ischemic stroke, who were admitted to our stroke center within 3 days, constituted the sample for sensitivity analysis; of these, 394 belonged to the floating population. Supplementary Table II indicates that between-group differences in baseline characteristics were similar to those observed in patients admitted within 1 day. Table 3 shows the impact of floating on patients with ischemic stroke admitted within 3 days. The impact on primary and secondary outcomes was consistent with that observed in our primary cohort, where we observed significantly higher chances of achieving favorable outcomes at 3 months in the floating population (OR: 0.62; 95% CI: 0.43–0.89, *P* = 0.009). However, significant interactive effects were found in the subgroups of smoking and drinking but not in the subgroup with minor stroke (*P* = 0.046, 0.005, and 0.081, respectively, Figure 4). The effect was more apparent in the floating population with no drinking habit (OR: 0.48; 95% CI: 0.32–0.72, *P* < 0.001) and that with no smoking history (OR: 0.49; 95% CI: 032–0.76, *P* = 0.001).

**Table 3:** The impact of floating on patients with subacute ischemic stroke (sensitivity analysis).

	**Indigenous population**	**Floating population**	***p*** **-value**	**Unadjusted**	***p*** **-value**	**Adjusted**	***p*** **-value**
	***N*** **= 502 (Reference)**	***N*** **= 394**		**OR**		**OR**	
**Primary outcome**							
Unfavorable outcome	137 (27.3%)	69 (17.5%)	**<0.001**	0.57 (0.41–0.78)	**0.001**	0.59 (0.40–0.86)	**0.007** ^**a**^
**Secondary outcome**							
Emergency medical service	161 (32.1%)	110 (27.9%)	0.19	0.82 (0.61–1.10)	0.179	0.93 (0.67–1.30)	0.682^b^
3-h arrival	173 (34.5%)	113 (28.7%)	0.071	0.76 (0.57–1.02)	0.066	0.79 (0.58–1.08)	0.145^b^
Endovascular therapy	85 (16.9%)	59 (15.0%)	0.46	0.86 (0.60–1.24)	0.429	0.84 (0.55–1.27)	0.399^b^

*OR, odds ratio; CI, confidence interval; Endovascular therapy included intravenous thrombolytic therapy and intravenous thrombectomy; In the multivariable logistic regression analysis, as for a: we adjusted age, gender, emergency medical service, prehospital delay, smoking history, drinking habit, NIHSS at admission, hypertension, diabetes, atrial fibrillation, dyslipidemia, intravenous thrombolytic therapy, intravenous thrombectomy, TOAST, and discharge mRS; as for b: we adjusted age, gender, smoking history, drinking habit, NIHSS at admission, hypertension, diabetes, atrial fibrillation, and dyslipidemia. Bold values mean statistically significant*.

## Discussion

To the best of our knowledge, this study is the first to find that in comparison with the indigenous population, the floating population with first-ever ischemic stroke was more likely to have a favorable outcome at 3 months. The use of EMS, 3 h arrival rate, and use of endovascular therapy were comparable between the indigenous population and the floating population in Shanghai.

With fast urbanization, China has been facing a unique problem of floating population in the past two decades. According to “China's floating population development report 2016” issued by the National Health and Family Planning Commission Mobile Population Service Center, the floating population size was ~253 million by the end of 2014, ~18% of the total population in China, which is considerably larger than most other social groups (16). A dominating majority of China's floating population comes from rural areas with low economic status, such as the Sichuan, Anhui, and Henan Provinces (1); this is consistent with the observation in the floating population of our study in Shanghai. It has been well-recognized in the literature that with poor working conditions, low socioeconomic status, and a unique health insurance system, the floating population is at a disadvantage with regard to healthcare (7–9, 17). Poorer healthcare may be the reason for the early onset of ischemic stroke among the floating population compared with that observed among the indigenous population of Shanghai.

The floating population in China shares some similarities with their counterparts—often referred to as “migrant workers” or “immigrants” —in other countries such as Canada and United States (18–21). Compared with the indigenous population, immigrants to high-income countries have a lower mortality rate and a lower incidence of cardiovascular disease, and this has been attributed to a “healthy migrant effect (22).” This is because of a selection bias whereby those in good health are more likely to decide to migrate than those with poorer health. Saposnik et al. (23) completed a population-based matched cohort study in Ontario, which indicated that new immigrants appear to be at a lower risk of premature acute stroke than the indigenous population. After adjusting for multiple confounders, the hazard ratio (HR) for stroke was 0.69 (95% CI: 0.64–0.74) for new immigrants. Similar risk estimates were also found in both ischemic and hemorrhagic stroke subtypes. Using data from the population-based Brain Attack Surveillance in Corpus Christi project, Hollenhorst et al. (24) found that long-term Mexican American immigrants displayed better stroke functional outcomes than non-immigrant Mexican Americans. However, after analyzing a population cohort in Ontario, Vyas et al. (21) found that although stroke care is similar in immigrants and indigenous population, new immigrants are more likely to be disabled at discharge (HR: 1.18; 95% CI: 1.13–1.22). The inconsistency in post-stroke outcomes may be caused by different destinations and patterns of stroke risk factors of immigrants. The definition of post-stroke outcome was also inconsistent among these studies.

In our study, we found nearly a 50% higher chance of achieving better outcomes in the floating population with ischemic stroke. The potential reasons why the floating population with ischemic stroke was more likely to achieve favorable short-term outcomes in comparison to the indigenous population were as follows. First, the floating population is younger and capable of competing with other young adults in big cities; therefore, these individuals would have a better outcome at 3 months after ischemic stroke. This effect is similar to the “healthy migrant effect.” Second, Shanghai is known for being an aging city with nearly 24% of the population aged more than 60 years. As shown in the baseline characteristics, the indigenous population with ischemic stroke was older than the floating population. Early onset of ischemic stroke among the floating population gave them higher chances of recovering from a stroke attack. Third, a phenomenon called “migrant return” was observed in the floating population (25), which means these individuals would return to their place of birth to rest or to be treated in the company of their family members when they had physical health problems. The change of surroundings around them and the relief from work stress could have been helpful in their recovery.

Interactive analysis indicated that only the floating population with healthy living habits (non-smoker, non-drinker) were more likely to have better outcomes at 3 months compared with the indigenous population, and this interaction still existed in our sensitivity analysis. Some explanations we deduced for this were as follows. First, younger age accompanied with healthy living habits could have significantly contributed to the favorable short-term outcomes. Second, the definition of smoking and drinking could also have played a role. In our study, people who quit smoking or drinking for more than 1 year were considered non-smokers or non-drinkers. As for the floating population with minor stroke, the interactive effect only existed among those admitted within 1 day, which indicates that prehospital delay limited the positive influence on the floating population. In light of these results, in-hospital secondary stroke prevention and early rehabilitation need to be more aggressive for the indigenous population with ischemic stroke, especially for those who self-report non-smoking or non-drinking status.

Many implications of our study need to be discussed. First, although the 3 h arrival rate between the floating population and indigenous population with ischemic stroke was comparable, the prehospital delay was longer for the floating population with a median time of more than 5 h. In-hospital stroke education and stroke educational sessions were necessary to be implemented in the floating population to shorten the time from symptom onset to hospital arrival. It is worth noting that the use of an ambulance was comparable between the two groups when they encountered stroke. Some may argue that a lower educational background among the floating population may limit the action of calling EMS. However, the determinant for the activation is always related to the severity of stroke as shown in previous studies (26, 27). The higher prevalence of diabetes among the indigenous population with ischemic stroke was probably because of the older age. In all, the prevalence of vascular risk factors and stroke subtypes was similar to that reported in other studies comprising the Chinese population (28, 29). With regard to the process of in-hospital care (thrombolysis and thrombectomy), it is inspiring to find that there were no significant differences between groups and the overall usage of endovascular therapy was similar to that specified in a recent China Stroke Statistic Report (30).

### Limitations

Several limitations merit consideration in this study. First, our study is an observational study. Although we adjusted for several confounders in the multivariable logistic regression analysis, variables that we have not included and those that cannot be assessed may still have an impact on our results. Second, there are significant differences in excluded and included patients. However, we achieved consistent results as shown in our primary cohort by conducting a sensitivity analysis with patients admitted within 3 days. Third, the definition of the floating population is slightly different from that reported in previous studies, which may limit the generalizability of our results. However, we believe it is an optimal and reliable way to spot those comprising the floating population. Regarding the duration of stay in Shanghai (some studies also referred to it as acculturation), studies have shown that it is related to the incidence and outcome of ischemic stroke (31–33). However, as we mentioned above, information related to this is not available in our dataset. It is worth investigating through a prospectively designed study since the impact of duration of stay in Shanghai on the outcome following ischemic stroke has never been investigated before. Fourth, this is a single-center study. Minhang district is only one of the 16 districts in Shanghai. In the mean-time, Shanghai is only one of the many first-tier cities in China. The floating population in Minhang district, Shanghai, may have different characteristics from districts in other cities. A prospective large-cohort study including floating population across China is needed in the future.

## Conclusion

Compared with the indigenous population, the floating population with the first-ever ischemic stroke was more likely to have a favorable outcome at 3 months. The use of EMS, 3 h arrival rate, and use of endovascular therapy were comparable between the indigenous population and the floating population in Shanghai.

## Data Availability Statement

The raw data supporting the conclusions of this article will be made available by the authors, without undue reservation.

## Ethics Statement

The study was approved by the Institutional Review Board Affiliated with Fudan University. The patients/participants provided their written informed consent to participate in this study.

## Author Contributions

XL: study concept and design, wrote the first draft, and revised the manuscript. QS: study design, wrote the first draft, revised the manuscript, and data verification. SY and JZ: data verification. HL: study concept and design, study supervision or coordination, and revised the manuscript. All authors contributed to the article and approved the submitted version.

## Conflict of Interest

The authors declare that the research was conducted in the absence of any commercial or financial relationships that could be construed as a potential conflict of interest.

## Publisher's Note

All claims expressed in this article are solely those of the authors and do not necessarily represent those of their affiliated organizations, or those of the publisher, the editors and the reviewers. Any product that may be evaluated in this article, or claim that may be made by its manufacturer, is not guaranteed or endorsed by the publisher.
