# Supplementary material for: Disparities in the Outcomes Following Ischemic Stroke Between the Floating Population and Indigenous Population of Shanghai
Source: Front Neurol. 2021 Dec 15;12:774337. doi: 10.3389/fneur.2021.774337 (PMC8715939; doi:10.3389/fneur.2021.774337)
Supplement: Supplementary file 1 [file Data_Sheet_1.docx]

**Online supplementary material**

**Figure I** Geographical distribution of floating population in Minhang district, Shanghai, China


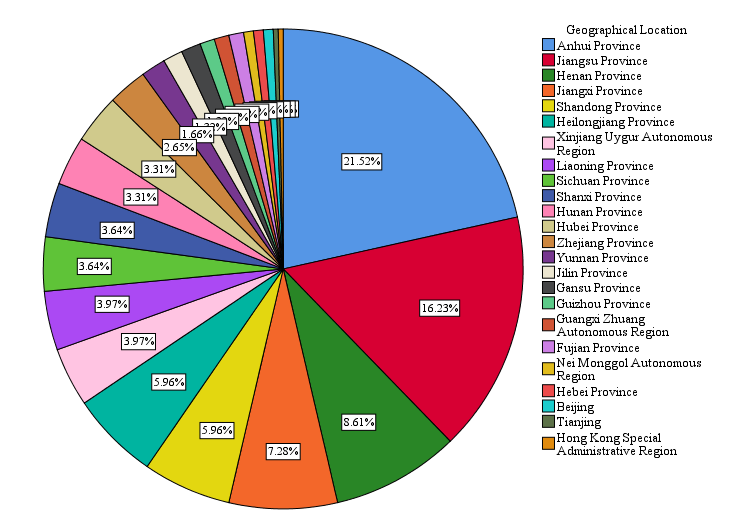


**Table I** Baseline characteristics of included and excluded patients in this study

| Factor | Included | Excluded |  |
| --- | --- | --- | --- |
| N | 698 | 2580 | P value |
| Age, mean (SD) | 65.32 (11.19) | 70.91 (12.60) | <0.01 |
| Gender (Male) | 521 (74.6%) | 1759 (68.2%) | <0.01 |
| Alcohol consumption | 97 (13.9%) | 220 (8.5%) | <0.01 |
| Smoking history | 213 (30.5%) | 450 (17.4%) | <0.01 |
| Medical histories |  |  |  |
| Hypertension | 463 (66.3%) | 1865 (72.3%) | <0.01 |
| Diabetes | 238 (34.1%) | 963 (37.3%) | 0.12 |
| Dyslipidemia | 196 (28.1%) | 548 (21.2%) | <0.01 |
| Atrial fibrillation | 81 (11.6%) | 324 (12.6%) | 0.52 |

**Table II** Baseline characteristics of patients with subacute ischemic stroke between the two groups (Sensitivity analysis)

| Factor | Floating population | Indigenous population | p-value |  |
| --- | --- | --- | --- | --- |
| N | 394 | 502 |  |  |
| Age, mean (SD) | 61.15 (11.66) | 68.18 (9.90) | <0.001 |  |
| Gender (Male) | 313 (79.4%) | 362 (72.1%) | 0.012 |  |
| Prehospital delay  (median, IQR, hour) | 8.13 (2.45, 22.95) | 6.28 (1.83, 19.63) | 0.043 |  |
|  |  |  |  |  |
| Alcohol consumption | 56 (14.2%) | 72 (14.3%) | 0.99 |  |
| Smoking history | 127 (32.2%) | 146 (29.1%) | 0.34 |  |
| Medical histories |  |  |  |  |
| Hypertension | 261 (66.2%) | 341 (67.9%) | 0.62 |  |
| Diabetes | 106 (26.9%) | 205 (40.8%) | <0.001 |  |
| Dyslipidemia | 118 (29.9%) | 137 (27.3%) | 0.41 |  |
| Atrial fibrillation | 31 (7.9%) | 59 (11.8%) | 0.058 |  |
| NIHSS at admission | 2 (1, 4) | 3 (1, 4) | 0.27 |  |
| Discharge mRS | 2 (1, 3) | 2 (1, 3) | 0.23 |  |
| Intravenous thrombolytic therapy | 55 (14.0%) | 74 (14.7%) | 0.77 |  |
| Intravascular thrombectomy | 12 (3.0%) | 17 (3.4%) | 0.85 |  |
| TOAST |  |  | 0.22 |  |
| Large-artery atherosclerosis | 146 (37.1%) | 200 (39.8%) |  |  |
| Cardioembolism | 22 (5.6%) | 44 (8.8%) |  |  |
| Small vessel disease | 186 (47.2%) | 206 (41.0%) |  |  |
| Other etiology | 12 (3.0%) | 13 (2.6%) |  |  |
| Unknown etiology | 28 (7.1%) | 39 (7.8%) |  |  |

Abbreviations: NIHSS: NIH Stroke Scale. IQR: interquartile range; P values represent the differences between indigenous population and floating population.
